# Patient-specific, echocardiography compatible flow loop model of aortic valve regurgitation in the setting of a mechanical assist device

**DOI:** 10.3389/fcvm.2023.994431

**Published:** 2023-02-08

**Authors:** Mahwash Kassi, Stefano Filippini, Eleonora Avenatti, Susan Xu, Kinan Carlos El-Tallawi, Clara I. Angulo, Marija Vukicevic, Stephen H. Little

**Affiliations:** ^1^Houston Methodist DeBakey Heart and Vascular Center, Houston Methodist Hospital, Houston, TX, United States; ^2^Department of Cardiology, Houston Methodist Research Institute, Houston, TX, United States; ^3^Department of Internal Medicine, Houston Methodist Hospital, Houston, TX, United States

**Keywords:** LVAD, aortic regurgitation, patient specific 3D printed phantoms, Doppler assessment, 3D printing

## Abstract

**Background:**

Aortic regurgitation (AR) occurs commonly in patients with continuous-flow left ventricular assist devices (LVAD). No gold standard is available to assess AR severity in this setting. Aim of this study was to create a patient-specific model of AR-LVAD with tailored AR flow assessed by Doppler echocardiography.

**Methods:**

An echo-compatible flow loop incorporating a 3D printed left heart of a Heart Mate II (HMII) recipient with known significant AR was created. Forward flow and LVAD flow at different LVAD speed were directly measured and AR regurgitant volume (RegVol) obtained by subtraction. Doppler parameters of AR were simultaneously measured at each LVAD speed.

**Results:**

We reproduced hemodynamics in a LVAD recipient with AR. AR in the model replicated accurately the AR in the index patient by comparable Color Doppler assessment. Forward flow increased from 4.09 to 5.61 L/min with LVAD speed increasing from 8,800 to 11,000 RPM while RegVol increased by 0.5 L/min (2.01 to 2.5 L/min).

**Conclusions:**

Our circulatory flow loop was able to accurately replicate AR severity and flow hemodynamics in an LVAD recipient. This model can be reliably used to study echo parameters and aid clinical management of patients with LVAD.

## Introduction

Continuous flow left ventricular assist device (cf-LVAD) technologies for end-stage heart failure patients have become a long-term treatment strategy (1). Development of *de novo* aortic regurgitation (AR) after implantation is a well-recognized complication of long term cf-LVAD support. One quarter to one-third of patients develop at least mild to moderate aortic regurgitation within the 1^st^ year and these patients face reduced device durability, higher rates of hospitalization, and worse survival (2, 3).

Current guidelines recommend surgical correction of more than mild AR at the time of LVAD implant but the treatment strategy of significant AR that develops after LVAD implant is more complex (4, 5). In this scenario, device-management with reduction of LVAD speed may be attempted to reduce the net-negative pressure created by the LVAD inflow cannula. In practice, it is not clear if decreasing the pump speed does decrease the AR severity or makes it worse. In addition, the accurate quantification of AR severity by Doppler methods is often very challenging in these patients. Guidelines for AR severity assessment in cf-LVAD patients recommend a multi-parametric approach based on traditional transthoracic echocardiography (TTE) parameters including pressure half time (PHT), vena contracta diameter (VC), jet width to left ventricular outflow tract width ratio, and corroboration with hemodynamic findings (6). However, these Doppler parameters have not been validated in this specific patient group with continuous suction from the inflow cannula of the LVAD to unload the LV. This results in continuous blood flow from the inflow cannula to the outflow cannula that is positioned in the proximal aorta (1, 6).

In this setting, the development of an *in vitro*, patient-specific, AR model that incorporates a cf-LVAD could represent a much-needed reference standard for dynamic flow and accurate AR volume quantification. Recently, 3D printing technology has been applied to the LVAD population for pre-implantation planning, but such patient-specific anatomic modeling has not been utilized for functional replication of this continuous flow/AR challenge (7–10).

The aim of the present study was to create a circulatory loop that combines 3D printed patient-specific geometry in order to: (1) replicate the hemodynamic conditions of AR in the presence of a cf-LVAD; (2) directly measure AR volume (RegVol) in an experimental setting to isolate the impact of LVAD speed changes on AR severity; and (3) assess the performance of recommended quantitative echocardiographic parameters for the assessment of AR severity compared to a reference standard of flow.

## Methods

### Patient-specific 3D printed model

For our study, we selected an 81-year-old male with HM II LVAD and a history of ischemic cardiomyopathy. Appropriate Institutional Review Board approval and consent were obtained from the patient.

**Figure 1:**
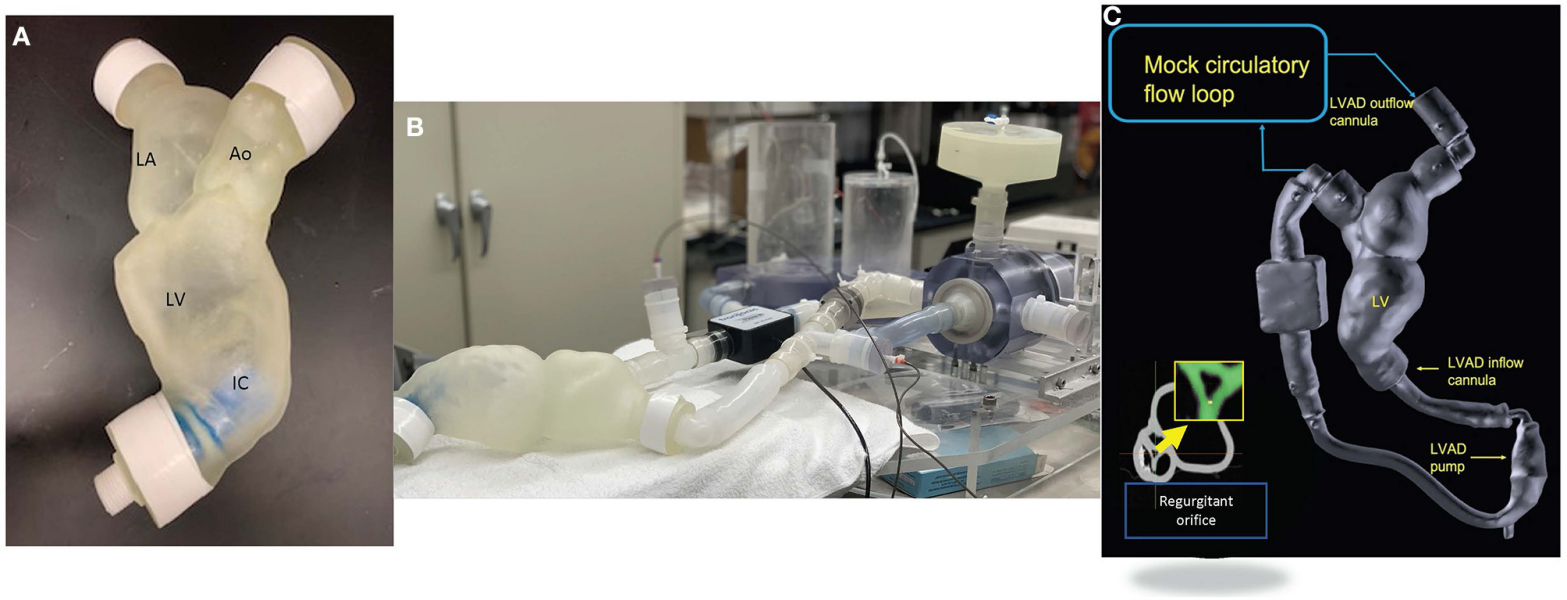
**(A)** Patient specific 3D printed model. **(B)** Mock circulatory flow loop setup. **(C)** Reconstruction from CT dataset of the patient specific model coupled with the Hearmate II LVAD and (inset) showing the regurgitate orifice. The yellow arrow indicates a zoom in on aortic regurgitation orifice.

From computed tomographic (CT) image data, segmentation of the patient-specific model was performed using Mimics software (Materialize NV, USA) (Figure 1A). The segmentation of the left heart was created based on pixel threshold intensity, including the inner region and the boundaries of the specific anatomic structures to be replicated (blood volume, left atrium, left ventricle, mitral valve, aorta, LVAD coupling connectors, and inflow cannula). The 3D digital model was then saved as a STL file and exported for 3D printing. Each anatomic element was 3D printed, considering the approximate mechanical properties of the biologic tissue and available elastomeric materials (Agilus shore, a Stratasys J750, Stratasys, 7665 Commerce Way Eden Prairie, MN 55344). The inflow cannula was also 3D printed to maintain a fixed inflow cannula position (Figure 1A). The aortic valve was configured to create a fixed regurgitant orifice area (ROA) of approximately 34 mm^2^ (Figure 1C inset). The material for the AV was chosen to replicate a stiff structure that would approximate the physiological finding in an elderly patient with calcified aortic leaflets.

We used a previously described process of segmentation to recreate a 3D patient-specific model. This model was outsourced for printing (11). Please see Supplementary Table 1 for details of the materials.

### Circulatory flow loop

**Figure 2:**
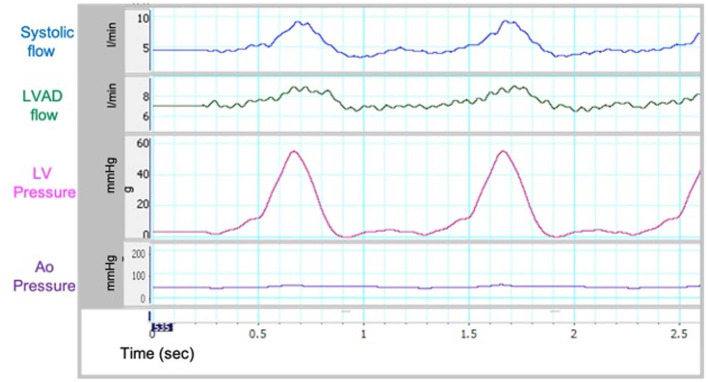
Flow and pressure curves from flow loop.

Our group has previously described an echo-compatible flow loop that, coupled with 3D printing technology, was able to replicate patient-specific hemodynamic conditions in different clinical settings, and provide reference standard flow and pressure measurements (12, 13). The flow loop was designed to achieve up to 7 L/min forward flow and provide variable compliance and resistance. Briefly, the loop consists of a pulsatile pump (Kollmorgen s300 brushless servo drive. Kollmorgen, Radford, V), arterial compliance and resistance elements, and a fluid reservoir (Figure 1B). High fidelity pressure transducers (Mikro-Tip Transducer, model SPR-370s. Millar, Houston, TX) were positioned on either side of the mitral valve and proximal to the aortic valve to record peak chamber pressure in the ventricle and the aorta. The pressure and flow information was recorded continuously (Figure 2). The pulsatile pump consists of a Lexan, hollow cylinder that houses a piston with an adjustable displacement volume up to 200 mL. Platinum cured silicone tubing was used to connect all flow loop elements. Beat-rate and flow conditions are controlled by a custom Labview virtual instrument (National Instruments, Austin, TX) program. For the present study, the flow loop configuration was designed to accommodate a HMII LVAD in the correct anatomical position. The flow loop was filled with a mixture of 30% glycerin, 70% water, and 0.01% cornstarch to simulate blood viscosity and ultrasound scattering behavior, as previously published (14, 15).

### Testing protocol and imaging

A fixed beat rate of 60 BPM and a fixed forward flow volume was used for all experimental conditions. With this constant preload, afterload and heart rate, 6 progressively higher LVAD speeds, from a baseline speed of 8,800 RPM to 1,100 RPM (8,800, 9,200, 9,600, 10,000, 10,400, and 11,000) were tested for their impact on aortic and left ventricular pressure, forward systemic flow and regurgitant flow. Doppler echo parameters were assessed at each LVAD pump speed.

For each LVAD speed setting, the systemic flow (L/min), cf-LVAD flow (L/min), aortic pressure (mmHg) and left ventricular pressure (mmHg) were directly captured. Aortic regurgitant volume (RegVol, ml) was calculated as the difference between the forward systemic flow measured directly within the flow loop through high fidelity transducers and cf-LVAD flow. An average of 3 measurements were recorded from the diastolic phase of 3 consecutive *in vitro* pulse cycles and used for statistical analysis.

### Echocardiography parameters

Echocardiographic acquisitions were performed using an IE33 machine (Philips, The Netherlands) equipped with a S5 probe for 2-dimensional and Doppler acquisition. Standard echocardiographic parameters for the assessment of AR severity [Continuous Wave Doppler on AR jet with evaluation of peak Velocity (cm/sec), Velocity Time Integral (VTI) and Pressure half time (PTH, msec); color Doppler of regurgitant jet width with operator appraisal, measurements of vena contracta (cm) and proximal isovelocity surface area (PISA, cm^2^)] and Pulsed Wave Doppler interrogation of inflow and outflow cannula for determination of systolic and diastolic velocities (cm/sec) were recorded for every flow condition. To minimize variability, echocardiographic acquisitions were standardized for gain, filters, compression and rejects settings. TTE parameters describing AR severity and PISA-derived regurgitant Volume (Vol_PISA, cc/beat) were compared to the reference standard represented by the RegVol measured within the flow loop as described above. To enhance image acquisition the left heart model was placed in a water filled bath. A modified apical view was used for all Doppler evaluations.

### Statistical analysis

Data were analyzed using STATA version 16 (StataCorp. 2019. Stata Statistical Software: Release 16. College Station, TX: StataCorp LLC). Continuous variables were evaluated for normality using the Shapiro-Wilk test for normal distribution. To explore the relationship among the different indexes of AR, the Pearson coefficient of correlation was tested with linear regression analysis. Repeatability of hemodynamic data was assessed with repeated measurements on a second set of experiment under the same conditions and quantified by direct Pearson's correlation. Inter-observer variability for echocardiographic data was assessed by repeated measurement by independent readers for all echo parameters and quantified by direct Pearson's correlation.

## Results

### Reproducing patient-specific hemodynamics

Sample waveforms for systemic and LVAD flow and left ventricular pressures in the flow loop are shown in Figure 2. These were similar to *in vivo* waveforms as reported by Rosenbaum et al. describing a left heart catheterization ramp protocol for hemodynamic optimization and variations in disease states (16). As such, our *in vitro* model was able to replicate the hemodynamic conditions of cf-LVAD recipients. The recorded pressure and flow within the circulatory loop were consistent on repeated measurements, with Pearson's correlation coefficients >0.96 for all analyzed Pressures and Flow variables (Supplementary Table 2).

### Hemodynamic parameters

**Figure 3:**
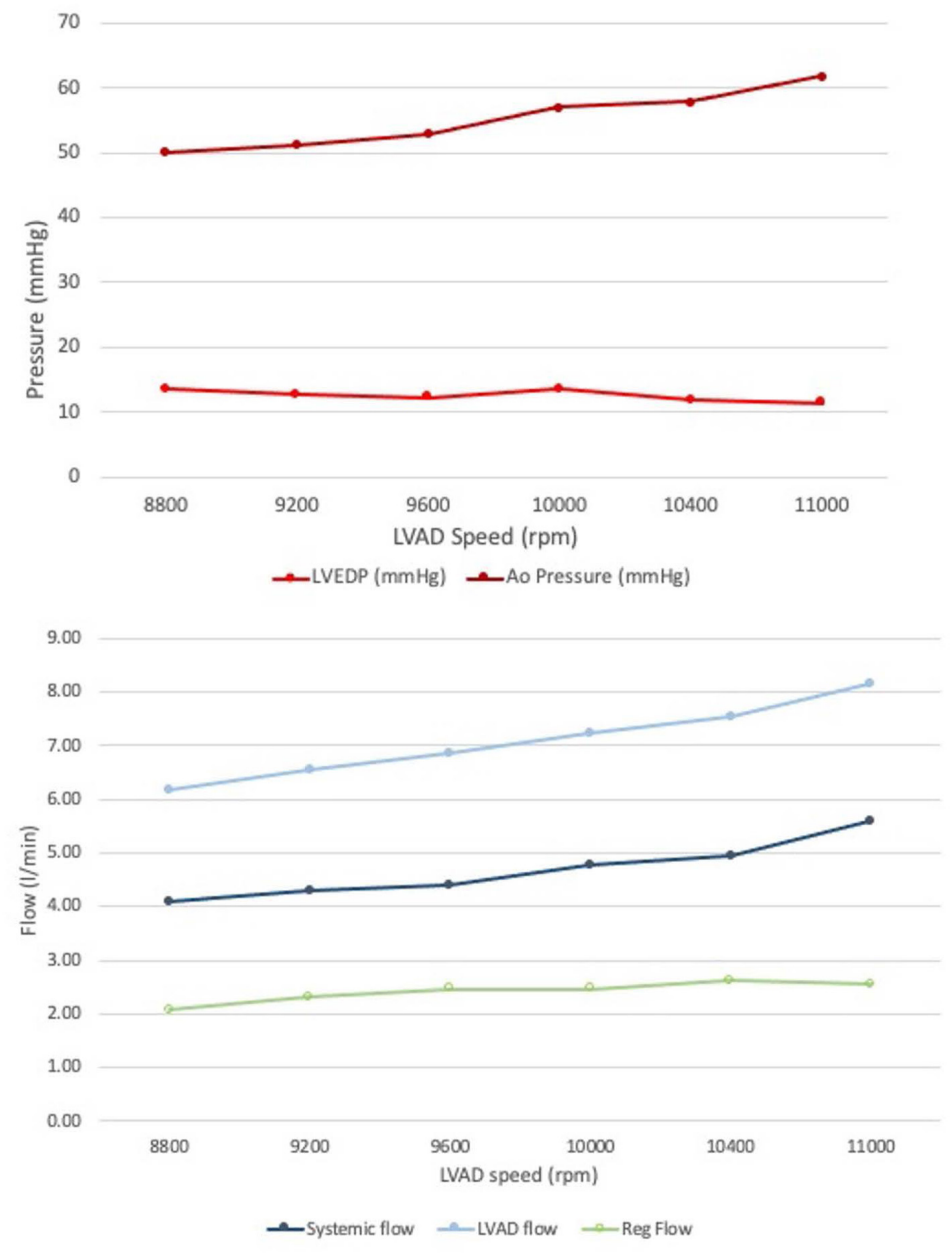
Invasive measurements obtained from the mock circulatory flow loop. **(Top)** End diastolic pressures in the Aorta and LV. **(Bottom)** Forward systemic flow and LVAD flow; regurgitant flow form AR obtained by subtraction: note how the regurgitant volume did not significantly change at increased LVAD speed.

As the LVAD speed increased from 8,800 to 11,000 RPM, forward flow increased from 4.09 to 5.61 L/min, which is in line with expected increased forward flow in the setting of increased left ventricular support provided by the cf-LVAD in clinical practice. Moreover, the mean systemic and aortic flow increased, and the end-diastolic aortic pressure increased, while the LV end-diastolic pressure (LVEDP) decreased, consistent with increased LV “unloading” with higher LVAD speed (Table 1, Figure 3).

**Table 1:** Directly measured hemodynamic parameters within the flow loop at different LVAD speeds.

	**Systemic flow**		**LVAD flow**		**Reg volume**		**LVEDP**		**Ao EDP**	
**LVAD SPEED** **(rpm)**	**l/min**	**% change**	**l/min**	**% change**	**l/min**	**% change**	**mmHg**	**% change**	**mmHg**	**% change**
8,800	4.09	–	6.17	–	2.07	–	13.52	–	36.5	–
9,200	4.29	4.8	6.56	6.37	2.31	11.69	12.66	−6.3	38.56	5.64
9,600	4.39	7.25	6.85	11.08	2.47	19.28	12.25	−9.4	40.7	11.5
10,000	4.79	17.11	7.25	17.45	2.46	18.7	13.48	−0.3	43.69	19.7
10,400	4.94	20.7	7.55	22.42	2.61	26.35	11.8	−12.7	46.16	26.5
11,000	5.61	32.25	8.16	32.25	2.54	22.9	11.35	−16.0	50.64	38.7

LVED, left ventricular end diastolic pressure; AoEDP, Aortic end diastolic pressure.

### Echocardiographic parameters

**Figure 4:**
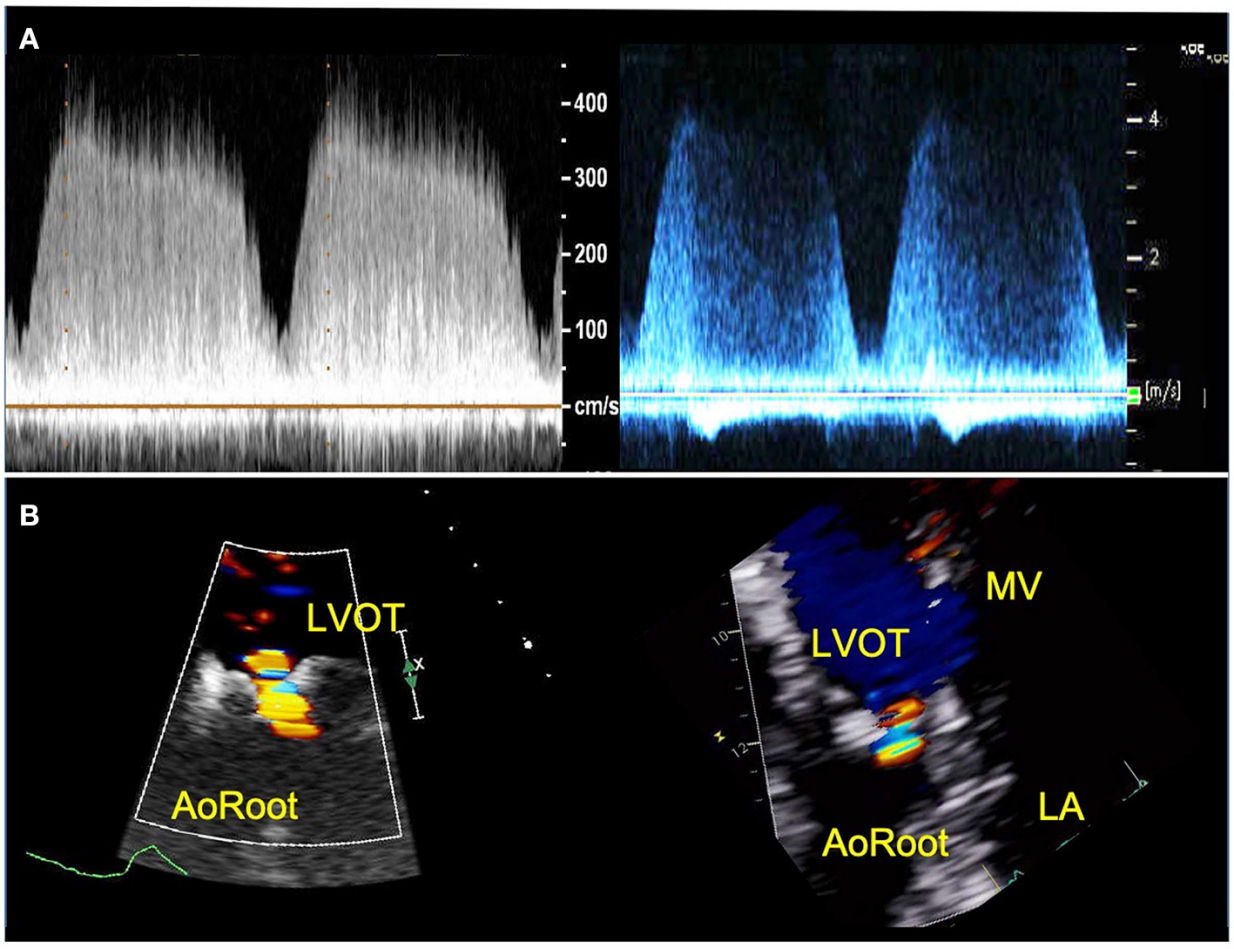
TTE images of AR from patient (right) and 3D printed model within the circulatory flow loop (left). **(A)** Continuous wave Doppler of AR flow demonstrating comparable profiles and peak velocities; **(B)** color Doppler images or the regurgitant AR flow, for vena contracta measurement. LVOT, Left ventricle outflow tract; LA, left atrium; MV, mitral valve.

AR created within the 3D patient-specific model replicated well the AR experienced by the patient with qualitatively similar continuous wave Doppler profile, peak velocities and event timing (Table 2, Figure 4). In addition, the color Doppler velocity map was very similar to the clinical echo depiction of AR severity.

**Table 2:** Impact of LVAD speed on echo parameters describing AR severity and echo based regurgitant volume calculation vs. directly measured AR regurgitant volume—image quality was deemed insufficient by both expert reader to obtain an accurate PISA radius and the subsequent calculations of ERO and RegVeol.

	**Echo parameters**								**Direct measurements**	
**LVAD SPEED**	**Vena contracta (cm)**	**PISA radius (cm)**	**VTI**	**Peak V (cm/sec)**	**PHT (msec)**	**S/D ratio**	**ERO (cm** ^2^ **)**	**Vol_PISA** **(cc/beat)**	**Reg volume (cc/beat)**	**SD** ^*^ **(cc/beat)**
8,800	0.6	—	247	355	700	1.348	—	—	34.53	1.19
9,200	0.75	0.4	248	362	820	1.354	0.12	31.66	37.94	1.13
9,600	0.8	0.7	274	385	870	1.232	0.27	75.77	41.11	0.79
10,000	0.9	0.8	283	387	894	1.251	0.36	101.69	40.94	0.51
10,400	0.92	0.9	287	393	985	1.171	0.45	128.53	43.61	1.18
11,000	1	0.95	289	400	1,180	1.178	0.49	141.68	42.50	0.50

* SD Standard deviation for repeated measures of direct measurements of Regurgitant volume within the flow loop.

### Effect of LVAD speed on AR severity

Increasing the LVAD speed, the RegVol increased only by approximately 0.5 L/min (2.01 to 2.5 L/min) or approximately < 10 ml/beat (34.5 to 42.4 ml/beat). As such, the severity remained moderate across all tested flow conditions.

**Figure 5:**
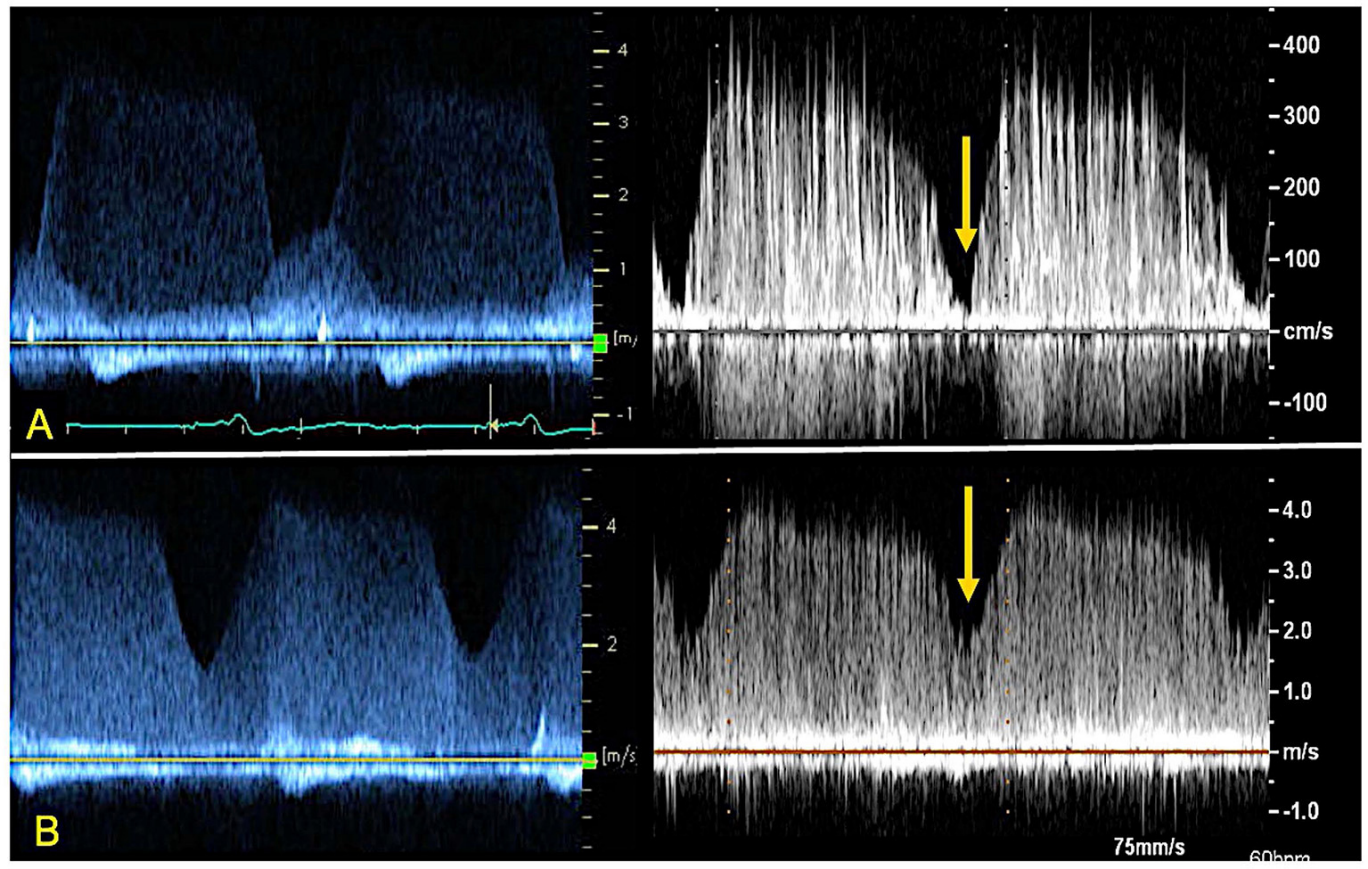
CWD from index patient's ramp study (left) and 3D printed model within the circulatory flow loop (right) depicting AR profile at different LVAD speeds. Note the loss of systolic dip in the CWD curve as the AR becomes “pancyclic” going from 8800 RPM **(A)** to 11000 RPM **(B)** of LVAD support. The yellow arrow indicates the “systolic dip”.

The evaluation of AR by continuous wave Doppler (CWD) demonstrated a trend toward increasing peak velocity (355 to 400 cm/sec) and regurgitant flow VTI (247 to 289) with increasing LVAD speed, pointing to a more significant AR. Moreover, a progressive increase in the systolic component of the flow was noted, that mirrored the pattern seen *in vivo* (the regurgitation tends to become more continuous or “pancyclic”, and loses the systolic pause, with regurgitant flow recorded in systole) with the increases of LVAD speed (Figure 5). PHT increased with increasing LVAD speed (700 to 1,180 ms), suggesting a direct correlation (R^2^ = 0.67) between PHT and RegVol that is inverse to what would be expected with worsening AR severity as suggested by higher peak velocities and AR VTI. Of note a similar pattern was seen in the *in vivo* echocardiographic clinical studies from the model patient (PHT from 1,175 to 1,865 ms with increasing LVAD speed from 8,800 to 10,600 RPM).

Color Doppler analysis revealed that with increasing LVAD speeds there was a progressive increase in vena contracta diameter (0.6 to 1 cm at 8,800 and 11,000 RPM respectively). As such, if the vena contracta diameter only appeared to classify the AR as moderate at 8,800 RPM, it reached measurements consistent with severe AR at higher LVAD speed. Similarly, a progressive increase in the PISA radius (0.4 to 0.94 cm) and thus PISA-derived regurgitation volume (AR_PISA, from 31.6 ml at 8,800 RPM to 141.7 ml at 11,000 RPM) was noted with increasing LVAD speed.

AR_PISA resulted in an overestimation that was progressively more substantial at increased LVAD speeds (+5 to +99 ml/min at 8,800 and 11,000 RPM respectively), and resulted in classifying AR as moderate at low LVAD speed and severe at higher RPM (≥9,600 RPM), even though as described the RegVol only changed by approximately 10 ml.

Peak systolic over peak diastolic velocity ratio (S/D) on pulse wave Doppler as measured at the outflow cannula a was measured for all flow conditions (17). The ratio remained <5.0 (range 1.36–1.24) and progressively decreased at increasing LVAD speeds. S/D inversely correlated with RegVol (R^2^ = 0.81) and its small range of variation was consistent with the small change in absolute RegVol.

The interobserver variability was good for all tested classic echocardiographic parameters, as showcased by high correlations for PHT, peak velocities, vena contracta diameter and PISA radius (R^2^ = 0.78, 0.95, 0.84 and 0.97 respectively for PHT peak velocity and vena contracta diameter). For S/D ratio the correlation was somewhat lower (R^2^ = 0.68).

Supplementary Figure 1 depicts association between echocardiographic parameters and directly measured regurgitant volume.

## Discussion

In this study we created a patient-specific flow model that allowed us to (i) accurately replicate AR in the setting of cf-LVAD and (ii) directly measure the regurgitant volume at different LVAD speeds. We present preliminary data demonstrating that changes in LVAD speed change AR severity by only a small fraction as based on directly measured regurgitant volume when other hemodynamic parameters remained constant, and traditional echocardiographic parameters overestimated the severity of AR in the cf-LVAD patient. Our findings support the current clinical guideline recommendations to avoid use of PHT to assess AR severity in LVAD patients and suggest that other Doppler profile elements such as AR duration and systolic flow interruption may be important for AR quantification (16).

In the current era of cf-LVAD, it is clearly established that AR is a common complication of long term LVAD use and is associated with worsening heart failure, poor end-organ perfusion, and decreased survival. Accurate quantification of aortic regurgitation remains difficult for cf-LVAD patients. Indeed, echocardiography lacks validation in this setting, and a reliable gold standard for regurgitant volume quantification is not clinically available. For instance, cardiac MRI cannot be performed due to the presence of a mechanical pump. Methods that combine echocardiography and right heart catheterization data to derive the aortic regurgitation volume have their own significant limitations (18).

In this study we describe an *in vitro* replication of dynamic flow conditions of cf-LVAD patients with AR that served as a gold standard against which standard TTE parameters for AR severity could be evaluated. Models simulating aortic regurgitation in cf-LVAD have previously been described. In a model of various regurgitant lesions by Shehab et al. they were able to successfully re-create the hemodynamic conditions of AR (19, 20). However, their work lacked a 3D patient specific model that was anatomically correct that allowed for accurate measurement of aortic valve area, left ventricular outflow tract and reproduced aortic valve regurgitation that could then be quantified by echocardiography.

Although our model incorporated a fixed-area aortic valve, the model behaves similarly to that of the aortic valve in an LVAD patient with minimal or no contribution to forward flow from the left ventricle. As such, we were able to test in isolation the effect the LVAD speed changes on the regurgitation volume and compare the direct measurements to the echo derived parameters of AR severity. Indeed, our experimental conditions maintained constant heart rate, preload, afterload, and the regurgitant valve area.

The net increase in the total regurgitation volume between a cf-LVAD baseline speed of 8,800 RPM and the maximum speed of 11,000 RPM was trivial in our experimental setting, somewhat surprisingly and in contrast to previous reports and our own echocardiographic findings (17, 21, 22).

This is helpful from a clinical perspective: knowing that the regurgitant volume does not increase significantly with LVAD speed is a significant argument against the common practice of attempting to mitigate AR severity through a reduction in LVAD support. Our experimental data suggests that the risk of inducing an increase in filling pressure and reduced cardiac output is not counterbalanced by a real impact on AR severity.

One change that did occur with increasing LVAD speed was to the time profile of AR on CWD analysis. In both the clinical echocardiogram and in our experimental setting, the CWD demonstrated diastolic AR for lower LVAD speed but pancyclic regurgitant flow with incremental speed. As mentioned however, the directly measured regurgitant volume (RegVol) did not change significantly. It can be therefore extrapolated that the AR is not always continuous but depends on LVAD speed and perhaps loading conditions. Therefore, we suggest that in clinical practice AR severity should be measured through TTE parameters at the lower LVAD speed.

In our study, the traditional parameter for severity, vena contract, and PISA overestimated AR severity. While these findings need further exploration, we have clearly demonstrated that PHT is unreliable for AR severity assessment. Indeed, we found an inverse relationship between PHT on CWD and AR severity. In our model, PHT progressively increased at increased LVAD speed—that is to say, for slightly increased RegVol, a trend that is contrary to what is normally expected in non cf-LVAD related AR. Current guidelines recommend against using PHT alone to grade AR severity, recognizing its dependence on LV preload, afterload and aortic pulse pressure. Of note, all of these are affected by the presence of the cf-LVAD, which creates a continuous flow in the aorta and presence of recirculating blood in the LV, making the PHT likely even less reliable to grade AR severity in this subset of patients.

As for the more recently proposed parameters to assess AR severity in cf-LVAD patients, the outflow cannula Doppler systolic to diastolic velocities ratio (S/D) remained <5.0 for all LVAD speed conditions in our model, correctly classifying AR as at least moderate (regurgitant fraction >30%) (17). The Doppler profile quality in our mock-circulatory flow loop did not allow for reliable measurement of diastolic acceleration, another proposed novel parameter for AR severity assessment, that was thus not tested. These new approaches will need further exploration.

Our preliminary data from this analysis, within the limitation of a 3D printed model, suggests that traditional echo-based approaches (vena contracta diameter and regurgitant volume by PISA method) significantly overestimate the AR severity and might thus represent a fallacious tool in guiding clinical management of this population especially at higher cf-LVAD speed. This is in stark contrast to common clinical practice and assumptions as well as more recent data that consider AR in cf-LVAD generally underestimated by echo parameters (17, 21, 22). This model can therefore be used in the future to test the echo parameters across a variety of different patient specific models.

## Conclusions

Our circulatory flow loop was able to closely replicate the AR flow and hemodynamics of a LVAD recipient, providing a gold standard of direct flow measures against which TTE-derived parameters of AR severity could be evaluated. Preliminary results indicate that with increasing LVAD speed, the increase in AR regurgitant volume is small, and that standard TTE parameters tend to overestimate such increase, more significantly so at higher LVAD support. Combined, these data might indicate the need for a critical rethinking of the application of traditional TTE parameters to guide the device management of *de novo* AR in patients with cf-LVAD. Further analysis will have to consider AR severity grading by other TTE parameters, as well as different patient specific 3D printed models and different LVAD devices.

## Limitations

We replicated and tested flow conditions with only one of the available cf-LVAD devices, the HeartMate II (Abbott, Chicago, IL); however, although is currently unclear whether incidence and impact of AR in cf-LVAD is dependent on the type of device, the majority of available data relate to the HMII.

The model replicated a small and constant contribution from the LV—provided in the model by the flow loop pump, that might not be the case for all cf-LVAD recipients but replicates the clinical scenario in which a residual LV function contributes to LVAD performances by augmenting VAD preload and providing some LVOT outflow. The right ventricle and pulmonary vasculature was not accounted for in the model, therefore the LV-RV interdependence as well as the effect on PA pressures could not be assessed. From an echo perspective, the model did not allow for insonation through a standard parasternal long axis view, therefore, a modified apical view was used for Doppler evaluation. Such a modified approach is however not uncommon in clinical practice, given the shadowing artifact produced by the LVAD inflow cannula. The 3D printed LV, although more compliant, still needs modification to simulate true diastolic function of the left ventricle.

The 3D-printing process was outsourced and material properties were not independently tested given the clinical focus of this study.

Finally, our model represents a single patient with heavily stiffened and remodeled aortic valve; further testing and modeling would be needed to confirm our findings on different patient specific modeling before fully being able to generalize our findings.

## Data availability statement

The raw data supporting the conclusions of this article will be made available by the authors, without undue reservation.

## Author contributions

MK, SL, and SF participated in the study design and conception. EA, CA, SF, and SX equally participated in the acquisition, analysis, and interpretation of data for the work. EA, MK, KE-T, and SL participated in the drafting of the work and its critical revision for important intellectual content. All authors provided approval for the final version of this manuscript and for the publication of the content and agreed to be accountable for all aspects of the work here presented.

## References

[1] Gasparovic H Kopjar T Saeed D Cikes M Svetina L Petricevic M. Novo aortic regurgitation after continuous-flow left ventricular assist device implantation. Ann Thorac Surg. (2017) 104:704–11. 10.1016/j.athoracsur.2017.01.114 28483150

[2] Fried JA Nazif TM Colombo PC. A new frontier for TAVR: Aortic insufficiency in CF-LVAD patients. J Heart Lung Transpl. (2019) 38:927–9. 10.1016/j.healun.2019.06.024 31495409

[3] Truby LK Garan AR Givens RC Wayda B Takeda K Yuzefpolskaya M. Aortic insufficiency during contemporary left ventricular assist device support: analysis of the INTERMACS registry. JACC Heart Failure. (2018) 6:951–60. 10.1016/j.jchf.2018.07.012 30384913 PMC6217859

[4] Feldman D Pamboukian SV Teuteberg JJ Birks E Lietz K Moore SA. The 2013 International Society for Heart and Lung Transplantation Guidelines for mechanical circulatory support: executive summary. J Heart Lung Transpl. (2013) 32:157–87. 10.1016/j.healun.2012.09.013 23352391

[5] Cowger J Rao V Massey T Sun B May-Newman K Jorde U. Comprehensive review and suggested strategies for the detection and management of aortic insufficiency in patients with a continuous-flow left ventricular assist device. J Heart Lung Transpl. (2015) 34:149–57. 10.1016/j.healun.2014.09.045 25511746

[6] Stainback RF Estep JD Agler DA Birks EJ Bremer M Hung J. American Society of Echocardiography. Echocardiography in the management of patients with left ventricular assist devices: Recommendations from the American Society of Echocardiography. J Am Soc Echocardiog. (2015) 28:853–909. 10.1016/j.echo.2015.05.008 26239899

[7] Farooqi KM Cooper C Chelliah A Saeed O Chai PJ Jambawalikar SR. 3D printing and heart failure: the present and the future. JACC Heart Failure. (2019) 7:132–42. 10.1016/j.jchf.2018.09.011 30553901

[8] Farooqi KM Saeed O Zaidi A Sanz J Nielsen JC Hsu DT. 3D printing to guide ventricular assist device placement in adults with congenital heart disease and heart failure. JACC Heart failure. (2016) 4:301–11. 10.1016/j.jchf.2016.01.012 27033018

[9] Saeed D Ootaki Y Noecker A Weber S Smith WA Duncan BW. The Cleveland Clinic PediPump: virtual fitting studies in children using three-dimensional reconstructions of cardiac computed tomography scans. ASAIO J. (2008) 54:133–7. 10.1097/MAT.0b013e31815b4495 18204330

[10] Karimov JH Steffen RJ Byram N Sunagawa G Horvath D Cruz V. Human fitting studies of cleveland clinic continuous-flow total artificial heart. ASAIO J. (2015) 61:424–8. 10.1097/MAT.0000000000000219 25806613 PMC4486514

[11] Vukicevic M Puperi DS Jane Grande-Allen K Little SH. 3D printed modeling of the mitral valve for catheter-based structural interventions. Ann Biomed Eng. (2017) 45:508–19. 10.1007/s10439-016-1676-5 27324801

[12] Little SH Igo SR Pirat B McCulloch M Hartley CJ Nosé Y. In vitro validation of real-time three-dimensional color Doppler echocardiography for direct measurement of proximal isovelocity surface area in mitral regurgitation. Am J Cardiol. (2007) 99:1440–7. 10.1016/j.amjcard.2006.12.079 17493476 PMC3348701

[13] Little SH Pirat B Kumar R Igo SR McCulloch M Hartley CJ. Three-dimensional color Doppler echocardiography for direct measurement of vena contracta area in mitral regurgitation: in vitro validation and clinical experience. JACC Cardiov Imag. (2008) 1:695–704. 10.1016/j.jcmg.2008.05.014 19356505 PMC3357190

[14] Valdes-Cruz LM Yoganathan AP Tamura T Tomizuka F Woo YR Sahn DJ. Studies in vitro of the relationship between ultrasound and laser Doppler velocimetry and applicability to the simplified Bernoulli relationship. Circulation. (1986) 73:300–8. 10.1161/01.CIR.73.2.300 2935326

[15] Jackson MS Igo SR Lindsey TE Maragiannis D Chin KE Autry K. Development of a multi-modality compatible flow loop system for the functional assessment of mitral valve prostheses. Cardiovasc Eng Technol. (2014) 5:13–24. 10.1007/s13239-014-0177-7

[16] de Marchi SF Windecker S Aeschbacher BC Seiler C. Influence of left ventricular relaxation on the pressure half time of aortic regurgitation. Heart. (1999) 82:607–13. 10.1136/hrt.82.5.607 10525518 PMC1760774

[17] Grinstein J Kruse E Sayer G Fedson S Kim GH Jorde UP. Accurate quantification methods for aortic insufficiency severity in patients with LVAD: Role of diastolic flow acceleration and systolic-to-diastolic peak velocity ratio of outflow cannula. JACC Cardiov Imag. (2016) 9:641–51. 10.1016/j.jcmg.2015.06.020 26684975

[18] Tehrani DM Grinstein J Kalantari S Kim G Sarswat N Adatya S. Cardiac output assessment in patients supported with left ventricular assist device: discordance between thermodilution and indirect fick cardiac output measurements. ASAIO J. (2017) 63:433–437. 10.1097/MAT.0000000000000528 28125464 PMC5489370

[19] Shehab S Allida SM Davidson PM Newton PJ Robson D Jansz PC. Right ventricular failure Post LVAD implantation corrected with biventricular support: an in vitro model. ASAIO J. (2017) 63:41–47. 10.1097/MAT.0000000000000455 28033201

[20] Shehab S Allida SM Newton PJ Robson D Macdonald PS Davidson PM. Valvular regurgitation in a biventricular mock circulatory loop. ASAIO J. (2019) 65:551–7. 10.1097/MAT.0000000000000852 30074964

[21] Estep JD Chang SM Bhimaraj A Torre-Amione G Zoghbi WA Nagueh SF. Imaging for ventricular function and myocardial recovery on nonpulsatile ventricular assist devices. Circulation. (2012) 125:2265–77. 10.1161/CIRCULATIONAHA.111.040238 22566350

[22] Bouabdallaoui N El-Hamamsy I Pham M Giraldeau G Parent M-C Carrier M. Aortic regurgitation in patients with a left ventricular assist device: A contemporary review. J. Heart Lung Transpl. (2018) 37:1289–97. 10.1016/j.healun.2018.07.002 30197211

