# Supplementary material for: Patient-specific, echocardiography compatible flow loop model of aortic valve regurgitation in the setting of a mechanical assist device
Source: Front Cardiovasc Med. 2023 Feb 8;10:994431. doi: 10.3389/fcvm.2023.994431 (PMC9945256; doi:10.3389/fcvm.2023.994431)

Supplement

| Structure | Material | Shore # |
| --- | --- | --- |
| Aortic valve | Agilus | 27 |
| Mitral valve | Agilus | 27 |
| Connectors | Agilus | 95 |
| Inner wall | Agilus | 27 |
| Shell | Agilus | 65 |

***Table 1S*** *Materials used for 3D printed patient specific model. All are Stratasys*

|  | Correlation Coefficient |
| --- | --- |
| Systemic flow | 0.9929 |
| LVAD flow | 0.9997 |
| Aortic pressure | 0.9976 |
| LVEDP | 0.9817 |
| Regurgitant Volume | 0.9612 |

***Table 2S*** *Correlation coefficients (Pearson's analysis) for repeated hemodynamic measurements within the flow loop*

Figure 1:

PHT:

| Model Fit Measures | | | | | | | |
| --- | --- | --- | --- | --- | --- | --- | --- |
|  |  |  |  |  |  |  |  |
| **Model** | | **R** | | **R²** | | **Adjusted R²** | |
| 1 |  | 0.819 |  | 0.671 |  | 0.589 |  |
|  | | | | | | | |

| Model Coefficients - PHT (msec) | | | | | | | | | | | | | | | | | | | |
| --- | --- | --- | --- | --- | --- | --- | --- | --- | --- | --- | --- | --- | --- | --- | --- | --- | --- | --- | --- |
|  | | | | | | **95% Confidence Interval** | | | |  | | | | | | **95% Confidence Interval** | | | |
| **Predictor** | | **Estimate** | | **SE** | | **Lower** | | **Upper** | | **t** | | **p** | | **Stand. Estimate** | | **Lower** | | **Upper** | |
| Intercept |  | -697.3 |  | 563.9 |  | -2262.80 |  | 868.3 |  | -1.24 |  | 0.284 |  |  |  |  |  |  |  |
| Reg Volume |  | 40.0 |  | 14.0 |  | 1.11 |  | 79.0 |  | 2.86 |  | 0.046 |  | 0.819 |  | 0.0226 |  | 1.62 |  |
|  | | | | | | | | | | | | | | | | | | | |


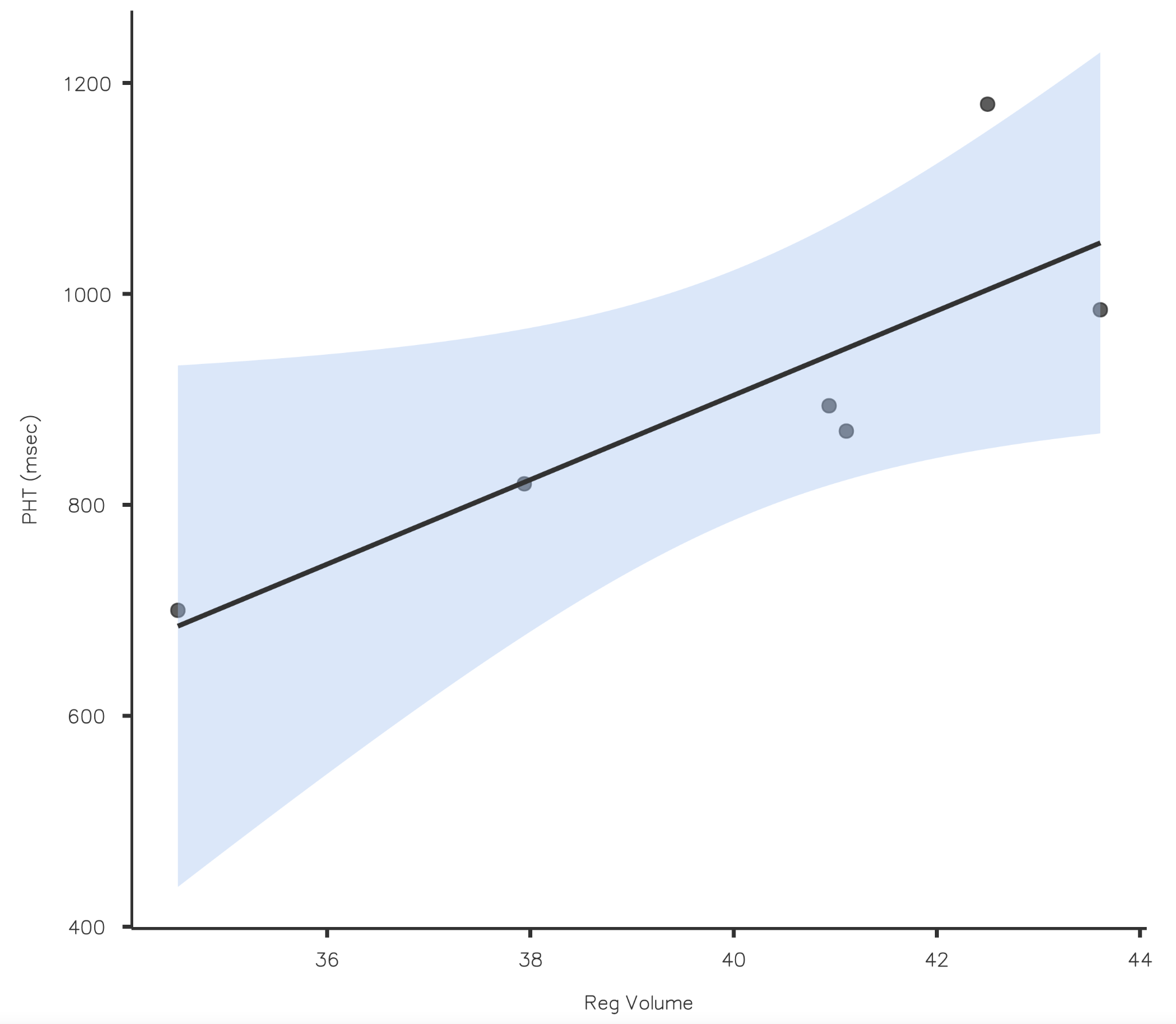


PISA Radium:

| Model Fit Measures | | | | | |
| --- | --- | --- | --- | --- | --- |
|  |  |  |  |  |  |
| **Model** | | **R** | | **R²** | |
| 1 |  | 0.943 |  | 0.890 |  |
|  | | | | | |

| Model Coefficients - PISA radius (cm) | | | | | | | | | | | | | | | |
| --- | --- | --- | --- | --- | --- | --- | --- | --- | --- | --- | --- | --- | --- | --- | --- |
|  | | | | | | | | | | | | **95% Confidence Interval** | | | |
| **Predictor** | | **Estimate** | | **SE** | | **t** | | **p** | | **Stand. Estimate** | | **Lower** | | **Upper** | |
| Intercept |  | -3.2227 |  | 0.8084 |  | -3.99 |  | 0.028 |  |  |  |  |  |  |  |
| Reg Volume |  | 0.0964 |  | 0.0196 |  | 4.92 |  | 0.016 |  | 0.943 |  | 0.333 |  | 1.55 |  |
|  | | | | | | | | | | | | | | | |


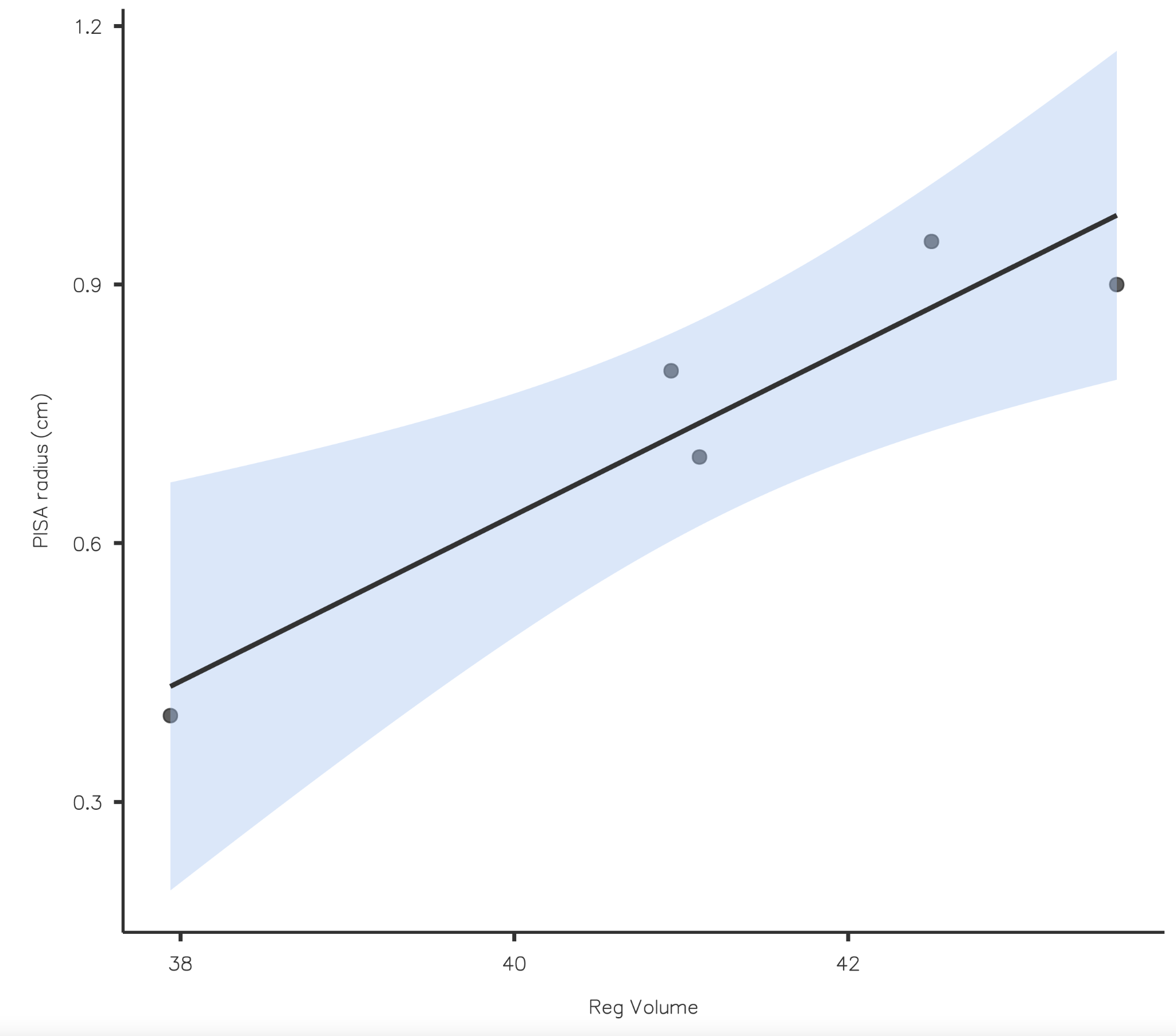


#
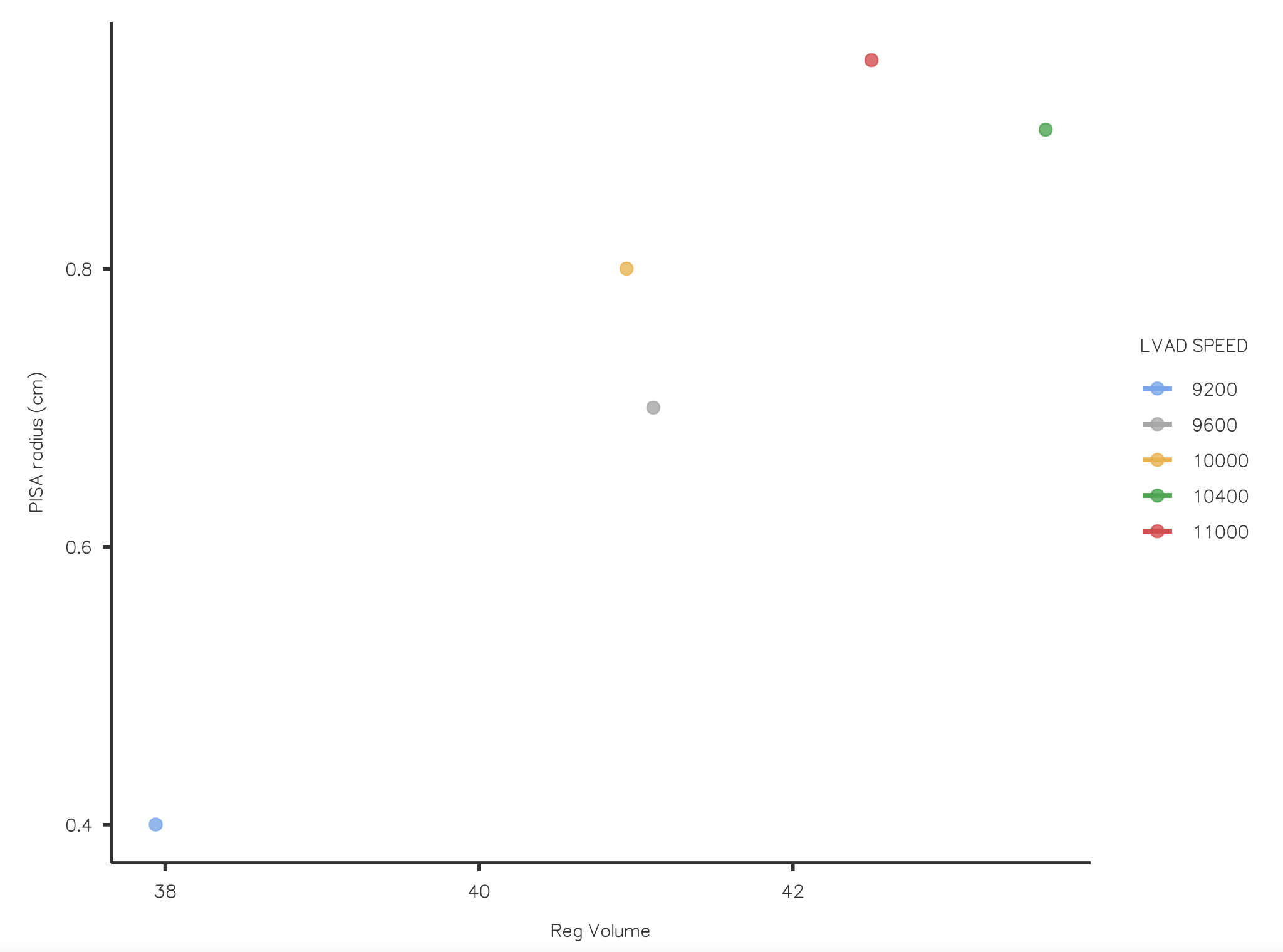


PISA volume:

| Model Fit Measures | | | | | | | |
| --- | --- | --- | --- | --- | --- | --- | --- |
|  |  |  |  |  |  |  |  |
| **Model** | | **R** | | **R²** | | **RMSE** | |
| 1 |  | 0.928 |  | 0.861 |  | 14.7 |  |
|  | | | | | | | |

| Model Coefficients - Vol_PISA | | | | | | | | | | | | | | | |
| --- | --- | --- | --- | --- | --- | --- | --- | --- | --- | --- | --- | --- | --- | --- | --- |
|  | | | | | | | | | | | | **95% Confidence Interval** | | | |
| **Predictor** | | **Estimate** | | **SE** | | **t** | | **p** | | **Stand. Estimate** | | **Lower** | | **Upper** | |
| Intercept |  | -692.1 |  | 183.20 |  | -3.78 |  | 0.032 |  |  |  |  |  |  |  |
| Reg Volume |  | 19.1 |  | 4.44 |  | 4.31 |  | 0.023 |  | 0.928 |  | 0.242 |  | 1.61 |  |
|  | | | | | | | | | | | | | | | |


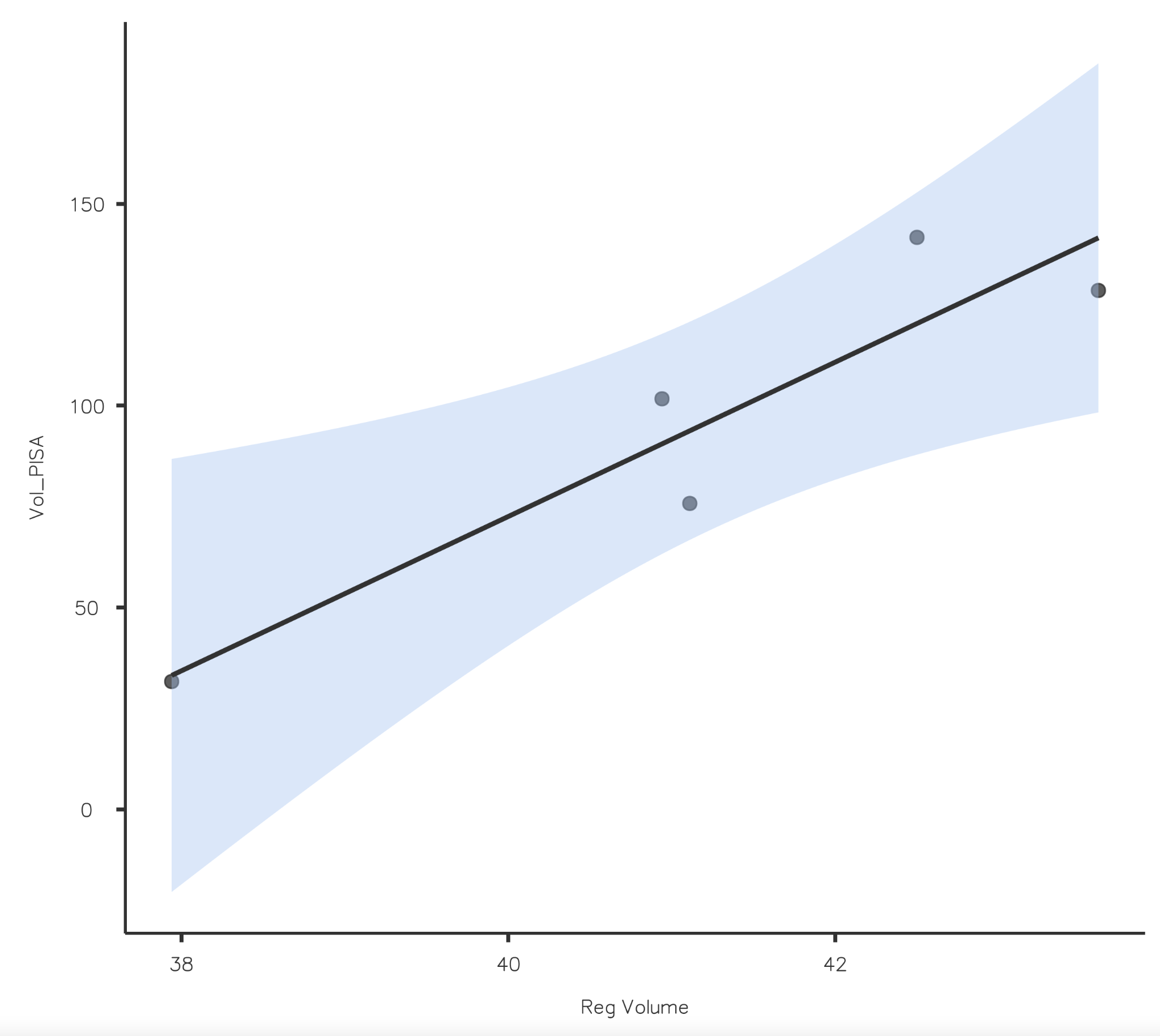


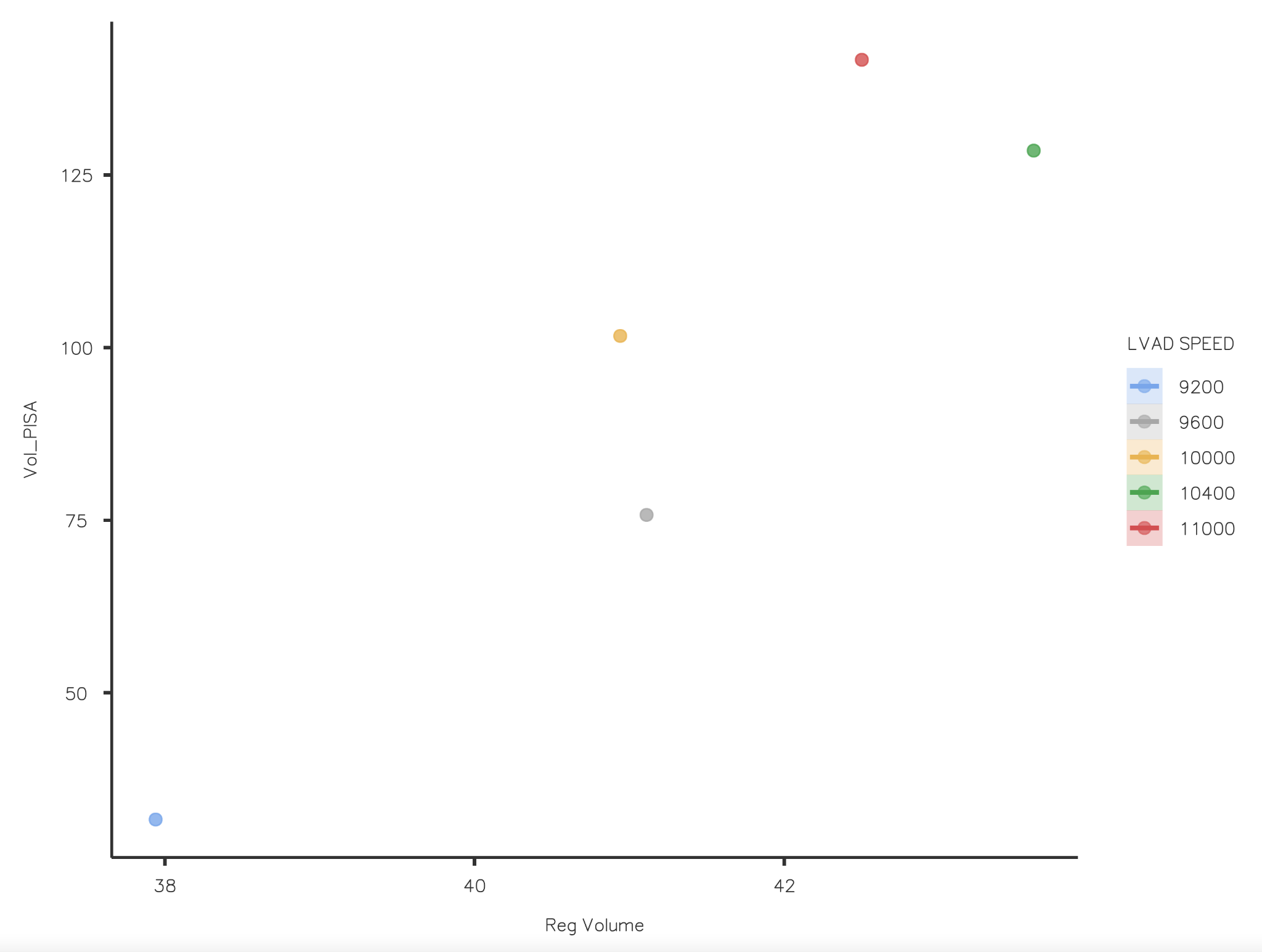


| Model Fit Measures | | | | | | | |
| --- | --- | --- | --- | --- | --- | --- | --- |
|  |  |  |  |  |  |  |  |
| **Model** | | **R** | | **R²** | | **RMSE** | |
| 1 |  | 0.927 |  | 0.859 |  | 0.0489 |  |
|  | | | | | | | |

| Model Coefficients - Vena contracta | | | | | | | | | |
| --- | --- | --- | --- | --- | --- | --- | --- | --- | --- |
|  |  |  |  |  |  |  |  |  |  |
| **Predictor** | | **Estimate** | | **SE** | | **t** | | **p** | |
| Intercept |  | -0.7661 |  | 0.32356 |  | -2.37 |  | 0.077 |  |
| Reg Volume |  | 0.0398 |  | 0.00804 |  | 4.94 |  | 0.008 |  |
|  | | | | | | | | | |


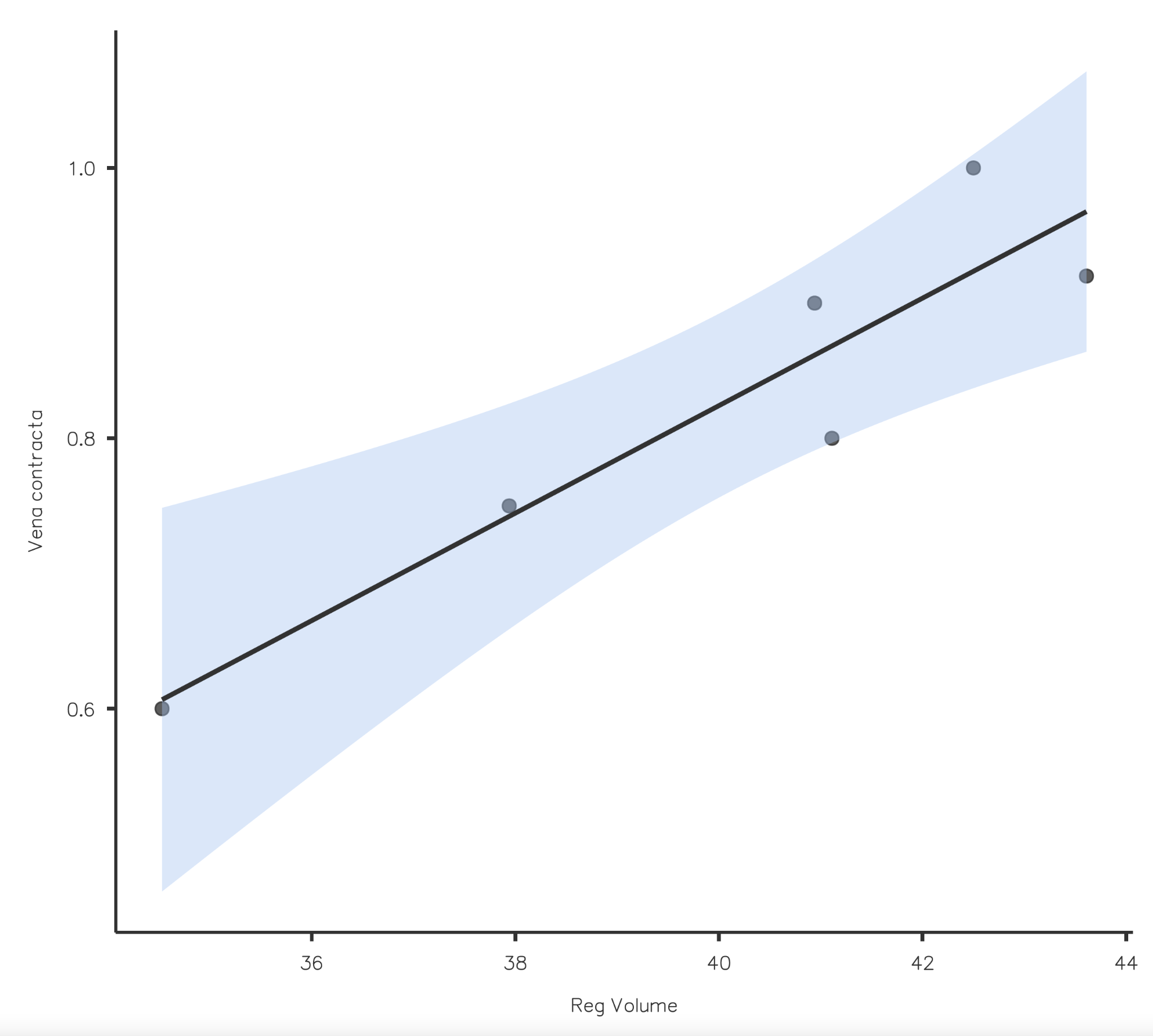

Supplement: Supplementary file 1 [file Data_Sheet_1.DOCX]
